# A comparison of rapid cycle deliberate practice and traditional reflective debriefing on interprofessional team performance

**DOI:** 10.1186/s12909-024-05101-1

**Published:** 2024-02-07

**Authors:** Nora Colman, Susan M. Wiltrakis, Sherita Holmes, Ruth Hwu, Srikant Iyer, Nandranie Goodwin, Claire Mathai, Scott Gillespie, Kiran B. Hebbar

**Affiliations:** 1 Department of Pediatrics, Division of Critical Care Medicine, Emory University School of Medicine, Atlanta, GA 30329 USA; 2 Department of Pediatrics, Division of Emergency Medicine, Washington University in St. Louis, 1 Children’s Place, St. Louis, MO 63110 USA; 3 Department of Pediatrics, Division of Emergency Medicine, Emory University School of Medicine, Atlanta, GA 30329 USA; 4 Children’s Healthcare of Atlanta, Atlanta, GA 30329 USA; 5 Scott Gillespie: Department of Pediatrics, Pediatrics Biostatistics Core, Emory University School of Medicine, Atlanta, GA USA

**Keywords:** Simulation-based team training, Interdisciplinary simulation, Rapid cycle deliberate practice, Traditional reflective debriefing

## Abstract

**Background:**

In simulation-based education, debriefing is necessary to promote knowledge acquisition and skill application. Rapid Cycle Deliberate Practice (RCDP) and Traditional Reflective Debriefing (TRD) are based in learning theories of deliberate practice and reflective learning, respectively. In this study, we compared the effectiveness of TRD versus RCDP on acquisition of conceptual knowledge and teamwork skills among interdisciplinary learners in the pediatric emergency department.

**Methods:**

One hundred sixty-four learners including emergency department attending physicians, fellows, nurses, medical technicians, paramedics, and respiratory therapists, participated in 28 in-situ simulation workshops over 2 months. Groups were quasi-randomized to receive RCDP or TRD debriefing. Learners completed a multiple-choice test to assess teamwork knowledge. The TEAM Assessment Tool assessed team performance before and after debriefing. Primary outcomes were teamwork knowledge and team performance.

**Results:**

Average pre-intervention baseline knowledge assessment scores were high in both groups (TRD mean 90.5 (SD 12.7), RCDP mean 88.7 (SD 15.5). Post-test scores showed small improvements in both groups (TRD mean 93.2 (SD 12.2), RCDP mean 89.9 (SD 13.8), as indicated by effect sizes (ES = 0.21 and 0.09, for TRD and RCDP, respectively). Assessment of team performance demonstrated a significant improvement in mean scores from pre-assessment to post-assessment for all TEAM Assessment skills in both TRD and RCDP arms, based on *p*-values (all *p* < 0.01) and effect sizes (all ES > 0.8). While pre-post improvements in TEAM scores were generally higher in the RCDP group based on effect sizes, analysis did not indicate either debriefing approach as meaningfully improved over the other.

**Conclusions:**

Our study did not demonstrate that either TRD versus RCDP was meaningfully better in teamwork knowledge acquisition or improving skill application and performance. As such, we propose Reflective Deliberate Practice as a framework for future study to allow learners to reflect on learning and practice in action.

**Supplementary Information:**

The online version contains supplementary material available at 10.1186/s12909-024-05101-1.

## Background

Simulation-based Team Training (SbTT) focuses on multidisciplinary education to impact patient safety through improved team performance [1–7]. Debriefing approaches can be described in two broad categories: deliberate practice and reflective debriefing, which both require adaptive expertise and facilitate transfer of learning [8–11]. Rapid Cycle Deliberate Practice (RCDP) is a framework for deliberate practice and performance feedback where immediate in-action application, and behavioral correction with a focus on ‘what’ the learner is doing builds muscle memory to hardwire skill application [1]. Facilitator directed pauses provide just-in-time feedback based on pre-identified learning objectives. Learners practice and repeat skills with immediate feedback until the pre-identified skill is effectively applied into clinical practice and workflow is achieved [3, 4, 12–14]. Mastery learning is achieved when the learner reaches the objective and can advance to the next phase. When not achieved, feedback and behavioral correction coupled with repetitive practice and immediate application helps learners meet objectives [1, 9].

In Traditional Reflective Debriefing, pre-determined objectives guide debriefing but the discussion is driven by learners. Here the focus of debriefing in on the rationale or the ‘why’ [1]. Learners explore their strengths and weakness through self-reflection and challenge their embedded assumptions to change their frames of reference, acquire new conceptual knowledge and modify their behavior [15]. TRD typically occurs as post-event debriefing with the rare opportunity for learners to apply what they learned in a repeated scenario [8]. PEARLS is a structured framework that guides facilitators to conduct TRD, including identification and closure of gaps in learner performance, knowledge, and skills [15].

There is a growing body of literature that studies the role of varying debriefing methodologies on learner knowledge and skill acquisition during simulation training [8, 12, 13, 16]. In our high acuity, quaternary pediatric children’s hospital, we have applied SbTT across multiple clinical areas to teach clinical teams communication skills and knowledge with the goal to optimize team performance and improve delivery of care. While team performance seemingly improves during simulation training, we have found that our teams struggle with consistent application of teamwork skills at the bedside during real emergency events despite perceived conceptual knowledge acquisition. The goal of our study was to analyze the impact of debriefing methodology on team performance measures in an effort to optimize utilization and maximize the impact of SbTT initiatives. We conducted a large-scale quasi-randomized comparative study of RCDP and TRD on the acquisition of teamwork knowledge and skills among interdisciplinary pediatric emergency department (ED) team members. We assessed the improvement in one group over the other in pre-to-post knowledge and team performance. As TRD promotes discussion and acquisition of conceptual knowledge without opportunity to practice skill application, we hypothesized that this debriefing methodology would only improve pre-to-post assessment of teamwork knowledge over RCDP. Since RCDP allows for deliberate practice, we hypothesized that this debriefing methodology would achieve improvement in pre-to-post assessment of team performance over TRD.

## Methods

### Trial design

**Fig. 1:**
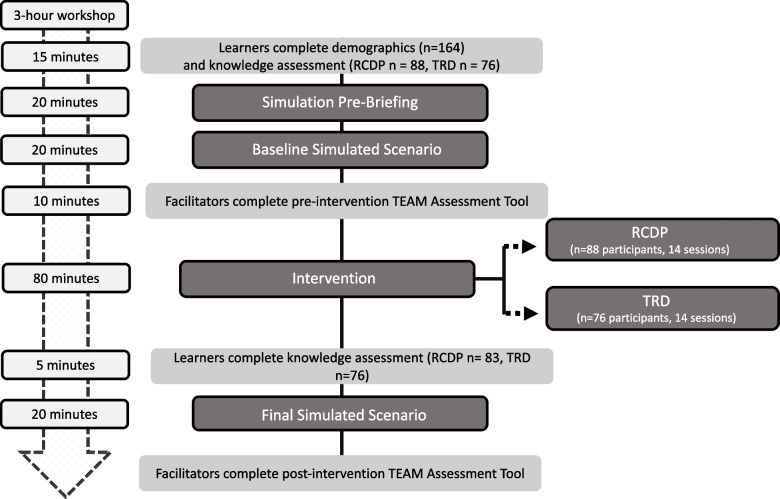
Flow diagram of Simulation-based Team Training and session timeline RCDP (Rapid Cycle Deliberate Practice) and TRD (Traditional Reflective Debriefing)

This was a prospective, un-blinded, parallel, quasi-experimental study, with a 1:1 allocation ratio, comparing RCDP to TRD debriefing (Fig. 1). Groups were quasi-randomized by the simulation team to RCDP or TRD debriefing based on date of the simulation workshop. Staff assigned themselves to a training workshop based on their schedule availability using an online scheduling platform. All Pediatric Emergency Medicine (PEM) staff (PEM attendings, PEM fellows, nurses, paramedics, medical technicians, and respiratory therapists) were eligable to participate in the study. There was no exclusion criteria. While participation in simulation was mandatory, participation in the research study was voluntary. All learners consented to study participation. Primary outcomes included teamwork knowledge and team performance before and after debriefing. This study was approved by our institution’s Institutional Review Board.

### Setting and participants

In-situ SbTT workshops simulating a child progressing from shock to cardiac arrest were conducted in the Egleston ED resuscitation room. The Egleston ED is one of three EDs within the Children’s Healthcare of Atlanta (CHOA) system. Egleston is a freestanding Children’s Hospital, with 80,000 ED visits. A high-fidelity human child mannequin (Gaumard Hal S157, 5 year old) with capabilities including heart and lung sounds, palpable pulses, and two functional intravenous (IV) lines were utilized. An electronic patient chart was created for the simulation. Simulation equipment embedded in the resuscitation room included IV fluids and tubing, mock code drug tray, defibrillator and pads, backboards, and airway equipment.

### Intervention

Twenty-eight workshops were conducted between October and November 2019. Each three-hour simulation workshop included the following learners: 1 team lead PEM attending, 1 PEM fellow to assist with intubation, 2–3 nurses, 2 paramedics or medical technicians and 1 respiratory therapist (RT). A simulation team (consisting of physicians, nurses, and RTs) facilitated scenarios.

### Scenario learning objectives

Teamwork learning objectives were identified based on three in-situ needs assessment simulations conducted in July and August 2019. During these simulations, frontline ED staff identified strengths and weaknesses in team performance during medical resuscitations. An ED guiding team (consisting of ED physician directors, nurse and RT managers, educators, and the simulation team) used this feedback to identify opportunities for improvement and design the training scenario. Specific teamwork learning objectives (Supplementary Table 1) were anchored to each phase of the scenario. The scenario was rehearsed with members of the ED guiding team to refine objectives and scenario progression.

### Briefing

Each simulation session began with a 20-minute briefing which introduced learners to the roles of participants, coaches and facilitators, their respective debriefing methodology, and session structure. RCDP learners were specifically briefed that they would be paused during the simulation to receive real-time feedback and that they would be prompted to repeat skills until mastery was achieved. Confidentially was established by informing all learners that individual performance would not be shared with unit leaders or educators and that all study data would be collected anonymously and not linked to individual learners. Learners were also oriented to the mannequin, resuscitation room, and supplies to be used.

Team roles necessary to conduct medical resuscitations were identified and clarified as part of the pre-briefing. These roles included the team lead physician, recording nurse, primary bedside nurse, medication nurse/pharmacist, RT, secondary intubating physician, medical technician, and/or paramedic. Nurses rotated in three different nursing roles (primary nurse, medication nurse, and documenter) to practice the learning objectives associated with those roles. The PEM attending maintained the role as the team lead physician while the PEM fellow was assigned to manage airway and assist the lead physician. Medical technicians, paramedics, and RTs maintained their roles throughout the training.

### Simulated scenario

In the scenario, a 6-year-old patient presented in shock. The patient developed respiratory failure and required endotracheal intubation. To meet testing objectives, the patient progressed to cardiac arrest, regardless of interventions made by the learners. Both RCDP and TRD groups were presented with the same clinical scenario. To standardize training, the scenario progression was pre-programmed, and a script with detailed learning objectives and pre-determined hard and soft stops were utilized for TRD and RCDP, respectively (Supplementary Table 1).

### Debriefing

A baseline simulation was conducted for both RCDP and TRD arms where the scenario was completed in entirety without any interuptions [1]. In the RCDP arm, multiple simulation cycles (RCDP intervention) were conducted in order to meet learning objectives. In the TRD arm, a single reflective debriefing occurred immediately after the baseline simulation. Following debriefing in both study arms, the learners conducted the scenario a final time without interruptions in order to incorporate and practice the skills and behaviors discussed (Fig. 1).

### Rapid cycle deliberate practice debriefing

Eighty minutes were allotted for RCDP pausing and coaching. Each subsequent phase of the scenario was more complex than the previous phase and anchored to new learning objectives. For example, a single phase focused on role clarity and role assignment, whereas the subsequent phase focused on directed and closed loop communication. The final phase (cardiac arrest) required learners to apply objectives from the prior phases (such as role assignment and closed loop communication) but also incorporated new skills training related to CPR performance.

RCDP was facilitated by a primary facilitator with extensive experience in application of RCDP for both algorithmic and teamwork training. Discipline specific feedback was provided by a nurse, physician, and RT coach. Coaches were context experts who supported the primary facilitator during debriefing [8]. Coaches enabled learners to extrapolate how feedback applied to them. For example, after the entire team was paused and coached on communication, the nurse coach provided feedback on application of closed loop and directed communication for medication administration. The RT coach then provided feedback to the RT learner on how the same skills applied to communication with the team leader regarding airway management. Feedback tailored to each discipline held the learner accountable for utilizing the skills that were being taught, suspending any assumptions that certain skills did not apply to them.

The facilitator and coaches observed the team for behaviors that either met or failed to meet the learning objectives. The primary facilitator paused the entire team to give feedback based on predetermined hard and soft stops. The nurse, physician, and RT coaches then provided discipline directed feedback. If the team successfully performed the learning objective, positive feedback was given. The facilitator rewound the scenario as many times as needed based on the teams’ ability to reach the hard stop objectives. The scenario progressed forward when the team successfully met the learning objective for each particular phase.

### Traditional reflective debriefing

The 80-minute debriefing session was conducted inside of the resuscitation room and immediately followed the baseline simulation. Debriefing followed the PEARLS framework. Sessions were guided by a facilitator with training and extensive experience in debriefing healthcare teams on team performance. Plus-delta strategies and advocacy-inquiry techniques reinforced successful team performance behaviors, identified gaps in team performance skills, assessed the learners’ frame and closed any performance gaps [15]. Facilitators focused on teamwork skills as the primary learning objective (Supplementary Table 1).

### Outcomes

Teamwork knowledge was assessed with a ten-question, multiple-choice test. This was completed by learners pre- and post-intervention in both study arms (Fig. 1). While not a validated instrument, the questions were based on prior knowledge assessments used in SbTT literature [14, 17]. Faciliators and coaches used the TEAM Assessment Tool to evaluate teamwork skills and team performance [17]. In a study to determine validity, reliability, and feasibility, Cooper et al. demonstrated uni-dimensional validity, concurrent validity, construct validity, and internal consistency [17]. The TEAM Assessment Tool can therefore effectively assess key skills necessary for effective emergency medicine team performance in the simulated environment and real clinical settings [17]. The tool is intended to rate the 11 items discretely and includes an overall team performance score based on a five-point Likert scale (0–4) [17]. Team performance was assessed after the baseline and final simulation (Fig. 1). To achieve interrater reliability, four raters, the primary facilitator and physician coaches, were trained in the application of the TEAM Assessment Tool. Specific examples of strong, average, and poor team performance were agreed upon by raters prior to the study. To avoid influencing the learners’ baseline teamwork knowledge, the multiple-choice test content and the TEAM Assessment Tool were not provided prior to simulation.

### Statistical methods

Demographics of the study participants were summarized using counts and percentages of TRD and RCDP participants. Differences between cohorts were evaluated using chi-square tests of independence or Fisher’s exact tests, based on expected frequencies.

Knowledge assessment was summarized at pre and post using means and standard deviations. Pre-post differences within cohorts were assessed using paired t-tests and effect sizes (ES). For our study, ES evaluated the standardized degree of improvement in outcomes between the pre and post assessments and were calculated by taking the mean pre-post differences within cohorts and dividing by the respective baseline standard deviations. ES were interpreted using Cohen’s *d* criteria, which consider the thresholds: small (0.2), moderate (0.5), and large (0.8). Differences in pre-post assessments between cohorts were evaluated by comparing thresholds of the within cohort ES. Results are reported as mean differences with 95% confidence intervals, *p*-values, and ES.

Statistical power was calculated by considering the mean change in TEAM ratings from pre to post assessment in each of the TRD and RCDP samples. A sample size of *N* = 15 participants provide at least 80% statistical power to detect an effect size of 0.78. Utilizing Cohen’s *d* criteria, this study had sufficient power to detect large changes from pre- to post-assessment within cohorts. Power was calculated using PASS v.14 (Kaysville, Utah), with a two-sided, paired t-test and a significance level of 0.05.

TEAM Assessment scores were summarized pre and post intervention using means, standard deviations, and paired t-tests. Results from the paired t-tests include mean differences from pre to post within study cohorts, standard errors, ES and *p*-values. Differences in pre-post assessments between study cohorts compared thresholds of the within cohort ES.

Inter-rater reliability was calculated for each TEAM Assessment question using intraclass correlations (ICC) and 95% confidence intervals. Four raters were included for analysis (facilitators and physician coaches), each session averaged 2–3 raters. ICCs were calculated using two-way random effects models, based on absolute agreement and single rater measurement. Due to rater imbalances, ICCs were bias-corrected using a generalized form of the two-way model proposed by Ebel [18]. All analyses were performed in SAS v.9.4 (Cary, NC) and CRAN R v.4.0 (Vienna, Austria). Statistical significance was evaluated at the 0.05 threshold.

## Results

Overall, 164 learners participated across 28 SbTT sessions SbTT study. There were no statistically significant differences in demographics between the TRD and RCDP groups (Table 1).

**Table 1:** Learner demographics

Characteristic	TRD *N* = 76	RCDP *N* = 88	P-Value
**Profession**			
Attending Physician	13 (17.1%)	14 (15.9%)	0.679
Fellow	2 (2.6%)	6 (6.8%)	
Medical Technician	16 (21.1%)	18 (20.5%)	
Nurse	34 (44.7%)	32 (36.4%)	
Paramedic	4 (5.3%)	5 (5.7%)	
Respiratory Therapist	7 (9.2%)	13 (14.8%)	
**Female Gender**	66 (86.8%)	71 (80.7%)	0.289
**Age (years)**			
18–29	29 (38.2%)	30 (34.1%)	0.290
30–39	30 (39.5%)	27 (30.7%)	
40–49	10 (13.2%)	21 (23.9%)	
50+	7 (9.2%)	10 (11.4%)	
**Time in Current Position (years)**			
< 2	17 (22.4%)	21 (23.9%)	0.411
2–5	30 (39.5%)	31 (35.2%)	
6–10	11 (14.5%)	13 (14.8%)	
11–15	7 (9.2%)	3 (3.4%)	
>15	11 (14.5%)	20 (22.7%)	
**Previous Simulation Training (years)**			
1–4	38 (50%)	48 (54.5%)	0.453
5–9	19 (25%)	15 (17.1%)	
>10	19 (25%)	25 (28.4%)	
**Prior Team-Based Training**	69 (90.8%)	71 (80.7%)	0.068
**Frequency of Medical Emergency Resuscitation Participation**			
Weekly	35 (46.1%)	39 (44.3%)	0.872
Every 2 Weeks	9 (11.8%)	11 (12.5%)	
Monthly	9 (11.8%)	15 (17.1%)	
Every Few Months	14 (18.4%)	13 (14.8%)	
Once a Year	7 (9.2%)	6 (6.8%)	
Never	2 (2.6%)	4 (4.5%)	

Knowledge post-test assessments were completed by 84% of learners in the TRD group, and 88% of learners in the RCDP group. Average pre-intervention baseline scores were high in both groups (TRD mean 90.5 (SD 12.7), RCDP mean 88.7 (SD 15.5). Post-test scores showed improvement in both groups (TRD mean 93.2 (SD 12.2), RCDP mean 89.9 (SD 13.8). This improvement in pre-post scores for TRD was statistically significant (Mean difference: 2.6, 95% CI: 0.04, 5.2, *p* = 0.047), but the improvement in pre-post scores for RCDP was not (Mean difference: 1.4, 95% CI: −0.9, 3.7, *p* = 0.227; ES = 0.09). Effect sizes, however, indicated improvements in both TRD and RCDP groups as small (ES = 0.21 and 0.09, respectively).

Assessment of team performance (Table 2) demonstrated a significant improvement in mean scores from pre to post assessment for all TEAM Assessment skills in both TRD and RCDP arms, as demonstrated by significant *p*-values (all *p* < 0.01) and large effect sizes (all ES > 0.8). While pre-post changes in TEAM scores were generally higher in the RCDP group based on ES (except Questions 4 and 6), these large ES did not indicate either debriefing approach as meaningfully improved over the other.

**Table 2:** Comparison of TEAM Assessment Scores

	Pre	Post	Post-Pre	Post-Pre	Bias-Adjusted Single Rater ICC
	Mean ± SD	Mean ± SD	Mean Difference (SE)	***P***-Value (ES)	(95% CI)
***Question 1: Directed Communication***					
**TRD**	1.64 ± 0.82	2.82 ± 0.77	1.18 (0.23)	< 0.001 (1.44)	0.520 (0.320, 0.680)
**RCDP**	1.30 ± 0.84	3.27 ± 0.50	1.97 (0.20)	< 0.001 (2.34)	
***Question 2: Global Perspective***					
**TRD**	2.00 ± 0.85	3.18 ± 0.46	1.18 (0.32)	0.003 (1.38)	0.458 (0.274, 0.620)
**RCDP**	1.87 ± 0.99	3.57 ± 0.56	1.70 (0.24)	< 0.001 (1.72)	
***Question 3: Effective Communication***					
**TRD**	1.18 ± 0.61	2.61 ± 0.63	1.43 (0.27)	< 0.001 (2.35)	0.563 (0.382, 0.708)
**RCDP**	1.10 ± 0.66	3.33 ± 0.52	2.23 (0.18)	< 0.001 (3.38)	
***Question 4: Timely Completion of Tasks***					
**TRD**	1.96 ± 0.50	3.29 ± 0.43	1.32 (0.17)	< 0.001 (2.67)	0.497 (0.316, 0.652)
**RCDP**	1.80 ± 0.68	3.57 ± 0.50	1.77 (0.15)	< 0.001 (2.62)	
***Question 5: Composure and Control***					
**TRD**	2.57 ± 0.58	3.11 ± 0.35	0.54 (0.16)	0.006 (0.92)	0.259 (0.064, 0.452)
**RCDP**	2.23 ± 0.86	3.47 ± 0.52	1.23 (0.23)	< 0.001 (1.44)	
***Question 6: Positive Morale***					
**TRD**	2.64 ± 0.41	3.36 ± 0.46	0.71 (0.18)	0.002 (1.74)	0.196 (0.009, 0.391)
**RCDP**	2.77 ± 0.78	3.47 ± 0.52	0.70 (0.18)	0.002 (0.90)	
***Question 7: Adaptation to Change***					
**TRD**	1.89 ± 0.59	2.93 ± 0.62	1.04 (0.22)	< 0.001 (1.75)	0.657 (0.510, 0.773)
**RCDP**	1.20 ± 0.75	3.40 ± 0.63	2.20 (0.19)	< 0.001 (2.93)	
***Question 8: Scenario Monitoring and Reassessment***					
**TRD**	2.36 ± 0.46	3.07 ± 0.51	0.71 (0.19)	0.003 (1.55)	0.495 (0.317, 0.650)
**RCDP**	1.80 ± 0.84	3.47 ± 0.64	1.67 (0.27)	< 0.001 (1.99)	
***Question 9: Anticipation of Actions***					
**TRD**	1.61 ± 0.71	3.14 ± 0.66	1.54 (0.27)	< 0.001 (2.15)	0.695 (0.553, 0.802)
**RCDP**	0.90 ± 1.11	3.30 ± 0.65	2.40 (0.25)	< 0.001 (2.17)	
***Question 10: Task Prioritization***					
**TRD**	1.00 ± 0.59	2.57 ± 1.04	1.57 (0.22)	< 0.001 (2.67)	0.470 (0.209, 0.663)
**RCDP**	0.93 ± 0.80	3.13 ± 0.90	2.20 (0.22)	< 0.001 (2.75)	
***Question 11: Followed Appropriate Guidelines***					
**TRD**	2.32 ± 0.70	3.36 ± 0.46	1.04 (0.23)	0.001 (1.49)	0.360 (0.165, 0.541)
**RCDP**	2.03 ± 0.67	3.53 ± 0.52	1.50 (0.20)	< 0.001 (2.25)	
***Question 12: Overall Teamwork Score***					
**TRD**	5.04 ± 1.20	7.68 ± 1.14	2.64 (0.44)	< 0.001 (2.20)	0.621 (0.466, 0.746)
**RCDP**	4.23 ± 1.45	8.43 ± 1.08	4.20 (0.30)	< 0.001 (2.90)	

Bias-Adjusted Single Rater Intraclass Correlation Coefficients (ICCs) were calculated for each TEAM Assessment question. The concepts with the highest rater agreement included anticipation of actions, adaptation to change, effective communication, and directed communication (ICC > 0.520)

## Discussion

This large interdisciplinary simulation study compared reflective learning versus deliberate practice to examine which debriefing methodology would be more effective in knowledge acquisition and performance. While we hypothesized that there would be a difference in knowledge acquisition and teamwork skills between the debriefing strategies, our results instead demonstrated the null effect. There was minimal improvement in the knowledge assessment due to ceiling effect. Both groups demonstrated improvement in team performance skills, and neither learning method was meaningfully better than the other.

In reflecting on our experiences with the two debriefing methodologies in this study, we noticed learners had a natural tendency to want to engage in the alternate debriefing approach at the completion of their session, indicating unique strengths in both methodologies. TRD learners expressed that there was insufficient opportunity to deliberately practice and apply the skills that were discussed during debriefing without practicing them correctly. At the learners’ request, facilitators provided feedback as they practiced closed loop communication during medication administration. In RCDP, learners did not have an opportunity to reflect on their practice, challenge their embedded assumptions, or reshape their frame of reference. RCDP learners discussed their own practice, explored relevant concepts, listened to perspectives of other team members, and discussed the rationale behind behavioral modifications. This highlighted the natural tendency for learners to want to reflect on their experience. While the pre-determined learning objectives were met, anecdotally it was evident that the simulation experience was incomplete without the opportunity for learners to reflect and practice. This experience suggests that reflection-in-action debriefing strategies should be considered. This concept has been elusively discussed by Eppich et al. who describes ‘microdebriefing’, where deliberate learning with feedback is coupled with reflection, yet the rationale, intuitional experience, and proposed approach is not robustly described [8].

In our experience, one clear benefit of TRD was that it facilitated a discussion between disciplines to level perceptions and suspend assumptions. This led to information sharing and improvement in communication, task completion, and task prioritization. As learners reflected on local culture, prior experiences, and perceptions, it was consistently unveiled that lack of a shared mental model and closed loop communication led to assumptions and misperceptions across disciplines. For example, staff was hesitant to interrupt the physician to read back and verify orders, close communication loops, and ask clarifying questions. It was perceived that communication directed at the team leader was disruptive. With further discussion during TRD, it was elucidated that the physician welcomed communication to reduce cognitive load and manage risk related to communication breakdown. TRD enabled learners to critically reflect and reframe their perceptions of hierarchy and information sharing [19].

The ability to apply new knowledge to practice was challenging because reflective learning did not allow for spontaneous skill application [9]. Learners reframed their perceptions and agreed that incorporation of teamwork skills into care delivery would benefit their teams’ performance, but they doubted if this would translate in practice. Issues related to poor team performance, such as insufficient resources and cultural nuances, were noted as latent threats that impeded clinical application. Conversely, RCDP learners overcame their own skepticism through application in practice. In contrast to reflective learning, deliberate practice allowed learners to master team performance behaviors through monitoring, error correction, and instantaneous feedback [4, 10, 16]. This was especially beneficial to practice skills that were referred to as “awkward”, “unnatural” or “redundant”. With focused, repetitive practice, learners self-adjusted and made improvements in role assignment, closed and directed communication and shared mental model before moving on to the next task. With practice, staff maintained their role assignments, spoke up when they were assigned to complete multiple tasks at once, asked clarifying questions, and provided feedback to the team leader.

Emerging literature on the impact of debriefing modalities on learner outcomes focuses on trainee learners. The debriefing modality that improves the performance of experienced providers is understudied [3, 14]. Unique to our study, learners included subspeciality trained PEM fellows and PEM attendings. Expert performers in other domains such as sports or music require continued deliberate practice to maintain skills mastery [11]. Yet, continued deliberate practice beyond organized medical training is underemphasized. Once advanced trained physicians adapt to their area of specialty, their skills become automated. Over time performance reaches a plateau and skill mastery deteriorates (arrested development) [10, 11].

During RCDP physicians required frequent error correction on role clarity, communication, and shared mental model to create new muscle memory and undo old behaviors, highlighting how difficult it was to incorporate new skills. Through deliberate practice, advanced trained physicians orchestrated a more organized and well-functioning team. Physicians can avoid arrested development associated with automaticity by engaging in Reflective Deliberate Practice. Here the rationale behind behavioral modification is elucidated through a shared experience that levels perception across disciplines and effective team leadership skills are mastered through deliberate practice.

Further research is needed, but next steps would include trialing a combined TRD and RCDP strategy, Reflective Deliberate Practice (RDP), a synergistic approach to debriefing. This could provide learners an opportunity to practice in action and reflect; a focus on the ‘what’ (RCDP) and ‘why’ (TRD) [1]. Reflective Deliberate Practice could challenge the learners’ frame is challenged during TRD and self-reflection is augmented through immediate course correction with repetitive deliberate practice. In Reflective Deliberate Practice, RCDP may be conducted with interval or a final cumulative reflective debriefing. Supplementary Table 2 provides an example of what training would look like if Reflective Deliberate Practice was applied (RCDP with interval TRD). Additional research comparing the application of single methodology debriefing versus Reflective Deliberate Practice is necessary to evaluate learner perception of each methodology, the impact of simulation training methodologies on skill acquisition and retention, and clinical application of skills beyond the simulated environment.

### Challenges and limitations

Studying the impact of simulation training on improved team performance is challenging. While many studies successfully demonstrate the impact of simulation on mastering algorithmic practices (adherence to algorithm/guidelines or time-to-event analysis), the ability to demonstrate team performance knowledge and skill acquisition and knowledge transfer beyond the simulated arena is much more challenging. For clinicians conducting in-situ simulations, where the goal is to address gaps in teamwork that are heavily influenced by the local micro-work system and unit specific culture, finding a tool that adequately aligns with specific training objectives is difficult. Previously validated tools either measure skills beyond the scope of the training or are too limited. Therefore, demonstrating a significant improvement in performance is challenging and fraught with limitations. Our study reflects these challenges as a ceiling effect and inadequate intra-rater reliability contributed to our inability to demonstrate meaningful significance.

The knowledge assessment was not a validated assessment tool, but the questions had been used in previous studies for similar learner groups [14]. High pre-test scores indicate a ceiling effect indicating that the questions were not powered to accurately discriminate change in knowledge in this population. This suggests a need to develop an assessment tool that is rigorous enough to identify knowledge gaps. Additionally, knowledge assessment scores were assessed retrospectively and not in real time. Therefore, learners did not receive immediate targeted feedback based on how they answered the questions. If assessed in real time, this information could be used as an adjunct during PEARLS debriefing to target learner deficiencies and close knowledge gaps. Due to time constraints, we did not assess retention of teamwork knowledge or skills following training, limiting the ability to demonstrate the impact of debriefing methodology on long-term skill retention.

Despite the TEAM Assessment Tool being validated, evaluation of team performance is subjective in nature, as these learning objectives are inherently non-algorithmic and pre-determined mastery standards for team performance skills do not exist, a limitation demonstrated in other studies [2, 3, 14]. When using these tools there is also a ceiling effect, where all learners demonstrated practice improvement. Additionally, this tool does not account for the impact that learner experience, perceptions, or local culture have on team performance and human driven behaviors. The ability to fully measure and assess skills such as composure and control is limited, as individuals’ emotions and cognitive load may not be fully recognized and explored by facilitators.

We attempted to achieve inter-rater reliability by limiting TEAM assessors to the same four individuals and having defined and agreed upon definitions for each category. However, all four raters were not present at every session nor was there full agreement on scores. Intraclass correlation coefficients were near or above 0.5 for 8 of 11 questions, but the more subjective TEAM questions (analyzing composure of team and morale) had lower agreement. Due to resource limitations, an independent observer was precluded from scoring team performance.

There are many challenges to establishing meaningful comparison and superiority data in simulation research. Resource limitations in tool choice, independent observers, number of learners to adequately power studies, and priority that focus on solving problems for clinical teams as opposed to the priority being research impact ability to conduct large studies that optimize research methodology. While this study was structured as a comparison study, future research may consider conducting inferiority studies.

## Conclusions

This large interprofessional simulation study that compared RCDP to TRD failed to demonstrate a clinically meaningful difference in team performance knowledge or skill acquisition improvements. Further research is needed to explore additional blended debriefing approaches, combining the strengths of TRD and RCDP to provide learners an opportunity to reflect and practice in action.

### Supplementary Information

**Additional file 1: Table S1.** Simulation scenario and learning objectives.

**Additional file 2: Table S2.** Example of Reflective Deliberate Practice; RCDP and interval TRD.

## Data Availability

The datasets used and/or analyzed during the current study are available from the corresponding author on reasonable request.

## Abbreviations

SBTT: Simulation-based Team Training
RCDP: Rapid Cycle Deliberate Practice
TRD: Traditional Reflective Debriefing
RDP: Reflective Deliberate Practice
PEM: Pediatric Emergency Medicine
ED: Emergency Department
RT: Respiratory Therapist

## Declarations

This study was approved by the Institutional Review Board at Emory University.
