# Supplementary material for: A comparison of rapid cycle deliberate practice and traditional reflective debriefing on interprofessional team performance
Source: BMC Med Educ. 2024 Feb 7;24:122. doi: 10.1186/s12909-024-05101-1 (PMC10848365; doi:10.1186/s12909-024-05101-1)
Supplement: Supplementary file 1 — Additional file 1: Table S1. Simulation scenario and learning objectives. [file 12909_2024_5101_MOESM1_ESM.docx]

| **Scenario Summary:**  6-year-old male with no past medical history, brought in by mother for vomiting and diarrhea or 24 hours, fever started this morning | | | |
| --- | --- | --- | --- |
|  | **TRD Learning Objectives**  ***(Time sensitive triggers)*** | **RCDP Learning Objectives** | |
| **Scenario State** |  | ***Hard Stops*** | ***Soft Stops*** |
| **I: Initial State**  Triage RN brings patient to the trauma room in a wheelchair. Triage RN calls charge RN informing them that the patient is being taken to the trauma room and asks them to page a medical emergency to assemble the team. Patient is tachycardic and hypotensive, consistent with shock. | ***(3 minutes)***  _Establishes that a medical emergency is needed  _Clarifies that all team member needed to manage a medical emergency are present | **RN:**  _Places patient on the monitor  _Communicates concern regarding arrival of unstable patient using SBAR  (Situation, Background, Assessment, Recommendation) | **RN:**  _Establishes that a medical emergency is needed |
| **II: Worsening Shock**  Patient develops worsening shock with progressive tachycardia and hypotension, requiring addition intravenous access, continued fluid resuscitation. Labs demonstrate metabolic acidosis and hypoglycemia requiring glucose administration. | ***(5 minutes)***  _Know my team  _Ensures that all team members needed are present  _Assign roles early and defines role responsibilities  _Define roles required for a medical emergency as soon as the team assembles. Roles include primary nurse, documenter, drug nurse, pharmacist, RT and secondary MD for intubation, paramedic or tech  _Role assignment  _Role Clarity  _Team member positioning | **Team:**  _Know my Team  _Team member role stickers  _Team member positioning  **MD:**  _Team leader is positioned at foot of bed  _Assigns roles  _Announces roles role to the whole team  _Establishes role clarity  **RN:**  _Uses code sheet  _Utilizes Broselow cart  **Medic/Tech:**  _Uses IV cart  _Uses closed loop communication  **RT:**  _Clarifies role | **MD:**  _Ensures that all team members needed are present  **RN:**  _Prompts role assignment and role clarity  _Verifies weight of patient  **Medic/Tech:**  _Shares mental model  _Directs information to the team leader |
| **III: Respiratory Failure requiring intubation**  Patient develops respiratory failure, characterized by poor respiratory effort, altered mental status, and hypoxia. Team initiates bag/mask ventilation, continues to provide fluid resuscitation, gathers equipment, supplies, and medications necessary for intubation. | ***(8-10 minutes)***  _Communication around medication preparation and administration  _Direct and closed loop communication  _Shared mental model | **MD:**  _Utilizes direct communication  _Utilizes closed loop communication  _Shares mental model  _Physician and nurse position themselves next to each other at the foot of the bed  **RN:**  _Read back and verification of medication orders  _Requests prioritization of medications/tasks  _Utilizes closed loop communication  _Utilizes directed communication  **RT:**  _Uses Broselow tray and pulls correct size airway adjuncts for intubation  _Utilizes closed loop communication  _Utilizes directed communication | **MD:**  _Maintains global assessment  _Re-establishes team leader identification  _Re-clarifies roles  _Directs second MD to intubate  **RN:**  _Maintains role assignment  **RT:**  _Communicates any needs with team leader  _Asks about potential for difficult airway |
| **IV: Pre-Intubation**  Team pauses just prior to intubation to review plan | ***(3-5 minutes)***  _Utilization of second physician for intubation  _Shared mental model  _Global Assessment  _Utilization of direct and closed loop communication | **MD Team Lead:**  _Shares mental model to discuss intubation risk assessment and plan  _Pauses team to reviews intubation Plan  **MD Intubator:**  _Reviews equipment and supply check list outload with entire team  _Shares mental model  **RT:**  _Shares mental model that intubation equipment is ready | **RN:**  _Asks any clarifying questions  _Maintains role assignment |
| **V: Cardiac Arrest**  Patient develops cardiac arrest during intubation requiring CPR | ***(6 minutes)***  _CPR skills  _Role clarity  _Global Assessment | **CYCLE 1**  _MD re-assigns and clarifies roles  _Medic/Tech places back board underneath patient  _Medic/Tech places Pads on patient  _Team uses metronome  _Compressor uses step stool  _Team coordinates compressions to breaths if not intubated  **CYCLE 2**  _Team members use direct and closed loop communication  _Team members maintain role assignment  **CYCLE 3**  **MD:**  _Monitors and provides feedback on CPR rate, recoil, depth  _Gives feedback to RT on bagging rate  _Doses epinephrine every 4 minutes  _During pulse Check coordinates rhythm check with change in compressors  _Verbalizes rhythm  _Anticipates next steps and shares with the team  **Recorder RN:**  _Scribe nurse stands next to physician at foot of bed and keeps track of two-minute CPR cycles  _Recorder gives summary to physician  _Verbalizes task completion  **Chest Compressor:**  _Counts out loud | **MD:**  _Encourages open exchange of ideas  _Maintains global assessment  _Provides step back and summary  **RN/RT**  _Recognizes change in vital signs and directs information to the team leader  _Maintains role assignment and clarifies roles  _Shares mental model  **Medic/Tech**  _Directs information to team leader |

Table 1. Simulation scenario and learning objectives

TRD: Traditional Reflective Debriefing, RCDP: Rapid Cycle Deliberate Practice, MD: Medical Doctor, RN: Nurse, RT: Respiratory Therapist, Medic: Paramedic, Tech: Technician
