# Supplementary material for: A comparison of rapid cycle deliberate practice and traditional reflective debriefing on interprofessional team performance
Source: BMC Med Educ. 2024 Feb 7;24:122. doi: 10.1186/s12909-024-05101-1 (PMC10848365; doi:10.1186/s12909-024-05101-1)
Supplement: Supplementary file 2 — Additional file 2: Table S2. Example of Reflective Deliberate Practice; RCDP and interval TRD. [file 12909_2024_5101_MOESM2_ESM.docx]

| Total Time: 3 hours  Pre-Brief: 30 minutes  Phase I: Shock 🡪 Respiratory Failure  RCDP: 40 minutes  TRD Phase I: 35 minutes | | Break: 5 Minutes  Phase 2: Respiratory Failure 🡪 Cardiac Arrest  RCDP: 35 minutes  TRD Phase II: 40 minutes  Wrap Up: 5 minutes | | |
| --- | --- | --- | --- | --- |
| **Phase I** | **RCDP Learning Objectives** | | | |
| **Scenario State** | ***Hard Stops*** | | | ***Soft Stops*** |
| **I: Initial State**  Triage RN brings patient to the trauma room in a wheelchair. Triage RN calls charge RN informing them that the patient is being taken to the trauma room and asks them to page a medical emergency to assemble the team. Patient is tachycardic and hypotensive, consistent with shock. | **RN:**  _Places patient on the monitor  _Communicates concern regarding arrival of unstable patient using SBAR  (Situation, Background, Assessment, Recommendation) | | | **RN:**  _Establishes that a medical emergency is needed |
| **II: Worsening Shock**  Patient develops worsening shock with progressive tachycardia and hypotension, requiring addition intravenous access, continued fluid resuscitation. Labs demonstrate metabolic acidosis and hypoglycemia requiring glucose administration. | **Team:**  _Know my Team  _Team member role stickers  _Team member positioning  **MD:**  _Team leader is positioned at foot of bed  _Assigns roles  _Announces roles role to the whole team  _Establishes role clarity  **RN:**  _Uses code sheet  _Utilizes Broselow cart  **Medic/Tech:**  _Uses IV cart  _Uses closed loop communication  **RT:**  _Clarifies role | | | **MD:**  _Ensures that all team members needed are present  **RN:**  _Prompts role assignment and role clarity  _Verifies weight of patient  **Medic/Tech:**  _Shares mental model  _Directs information to the team leader |
| **III: Respiratory Failure requiring intubation**  Patient develops respiratory failure, characterized by poor respiratory effort, altered mental status, and hypoxia. Team initiates bag/mask ventilation, continues to provide fluid resuscitation, gathers equipment, supplies, and medications necessary for intubation. | **MD:**  _Utilizes direct communication  _Utilizes closed loop communication  _Shares mental model  _Physician and nurse position themselves next to each other at the foot of the bed  **RN:**  _Read back and verification of medication orders  _Requests prioritization of medications/tasks  _Utilizes closed loop communication  _Utilizes directed communication  **RT:**  _Uses Broselow tray and pulls correct size airway adjuncts for intubation  _Utilizes closed loop communication  _Utilizes directed communication | | **MD:**  _Maintains global assessment  _Re-establishes team leader identification  _Re-clarifies roles  _Directs second MD to intubate  **RN:**  _Maintains role assignment  **RT:**  _Communicates any needs with team leader  _Asks about potential for difficult airway | |
| **TRD Learning Objectives**  **Facilitator Directed Objectives**  _Discusses what team member roles are necessary to manage a medical resuscitation  _Use of Broselow tray for intubation  _Use of a pause or time-out prior to intubation  _Utilization of a second physician for intubation  **Learner Directed Objectives**  _Know my team  _Role Assignment  _Role Clarity  _Team member positioning  _Closed loop communication  _Directed communication  _Shared mental model  ** If conducting RCDP with a cumulative TRD (as opposed to interval TRD described here), remove this step and combine learning objectives into the final debriefing following Phase II | | | | |
|  | | | | |
| **Phase II** | ***Hard Stops*** | | ***Soft Stops*** | |
| **IV: Pre-Intubation**  Team pauses just prior to intubation to review plan | **MD Team Lead:**  _Shares mental model to discuss intubation risk assessment and plan  _Pauses team to reviews intubation Plan  **MD Intubator:**  _Reviews equipment and supply check list outload with entire team  _Shares mental model  **RT:**  _Shares mental model that intubation equipment is ready | | **RN:**  _Asks any clarifying questions  _Maintains role assignment | |
| **V: Cardiac Arrest**  Patient develops cardiac arrest during intubation requiring CPR | **CYCLE 1**  _MD re-assigns and clarifies roles  _Medic/Tech places back board underneath patient  _Medic/Tech places Pads on patient  _Team uses metronome  _Compressor uses step stool  _Team coordinates compressions to breaths if not intubated  **CYCLE 2**  _Team members use direct and closed loop communication  _Team members maintain role assignment  **CYCLE 3**  **MD:**  _Monitors and provides feedback on CPR rate, recoil, depth  _Gives feedback to RT on bagging rate  _Doses epinephrine every 4 minutes  _During pulse Check coordinates rhythm check with change in compressors  _Verbalizes rhythm  _Anticipates next steps and shares with the team  **Recorder RN:**  _Scribe nurse stands next to physician at foot of bed and keeps track of two-minute CPR cycles  _Recorder gives summary to physician  _Verbalizes task completion  **Chest Compressor:**  _Counts out loud | | **MD:**  _Encourages open exchange of ideas  _Maintains global assessment  _Provides step back and summary  **RN/RT**  _Recognizes change in vital signs and directs information to the team leader  _Maintains role assignment and clarifies roles  _Shares mental model  **Medic/Tech**  _Directs information to team leader | |
| **TRD Learning Objectives**  **Facilitator Directed Objectives**  _CPR Skills  _Use of a pause or time-out prior to intubation  _Utilization of a second physician for intubation  **Learner Directed Objectives**  _Closed loop communication  _Directed communication  _Role Assignment  _Role clarity  _Global assessment  _Shared mental model | | | | |

Table 2. Example of Reflective Deliberate Practice; RCDP and interval TRD

TRD: Traditional Reflective Debriefing, RCDP: Rapid Cycle Deliberate Practice, MD: Medical Doctor, RN: Nurse, RT: Respiratory Therapist, Medic: Paramedic, Tech: Technician
